# The Marriage of the Unfit*Founded upon a paper read before the Watford Medical Society on April 1, 1898.

**Published:** 1898-10

**Authors:** Harry Campbell

**Affiliations:** M. D., B. S. Lond., F. R. C. P. Lond., Physician to the Northwest London Hospital; Wimpole Street, W.


### The Marriage of the Unfit.*

HARRY CAMPBELL, M. D., B. S. LOND., F. R. C. P. LOND., PHYSICIAN TO THE NORTHWEST LONDON HOSPITAL.

The fitness of two individuals to marry embraces two considerations—their fitness as regards each other and their fitness as regards the production and rearing of offspring. Fitness of the former kind implies mental suitableness and also, where there are no private means, the ability of the one or the other to make the necessary provision; except when this is necessary physical health, desirable though it may be, is not an essential. There may be a "marriage of true minds" without it. It is not this kind of fitness which I propose to discuss, nor the ability of parents to rear children—i. e., properly to provide for and educate them, which is a separate question altogether and a very wide one—but rather the fitness to produce children who shall reach a certain level of mental and bodily excellence.

When men come to understand the potency of heredity the question of marital fitness will receive more attention, and they

*Founded upon a paper read before the Watford Medical Society on April 1, 1898.

will begin to realize that the responsibility of making a life is scarcely less tremendous than that of taking it. As it is, one of the last things that occur to a marrying couple is, whether they are fit to be represented in posterity. I know of few things more remarkable than the extreme self-complacency of human beings (and these often the most unfit) in this respect. Yet even as things now stand the physician is not infrequently called upon to decide whether individuals are fit as regards the production of children, and in view of the enormous responsibitity thus thrown upon him it behooves him to be ready with a well-formed judgment in such cases. That they will come before us more and more in the future is to be both hoped and expected, and it is with the view to help to create a proper public opinion on these matters that I bring this subject before the profession.

In order that we may gain a philosophic insight into our subject we must understand the mode in which the human race is evolving and the part which heredity plays in shaping structure and causing disease.

We have still much to learn concerning the factors which determine animal evolution, but almost all are agreed that the principle of the "survival of the fittest" plays a predominant part in it. While it is a fact that the environment tends to mould the organism adaptively, in such a way, namely, that the latter becomes adapted to the environment, it seems very doubtful whether the direct action of the environment plays a decided part in animal evolution, because environment mouldings (as we may term them) do not appear to be inherited. At any rate, no unmistakable instance of the inheritance of an acquired character has yet been observed. It is therefore probable that organic evolution does not take place, or takes place only in a very slight degree, by a summation through successive generations of environmental mouldings. I need scarcely say how unwillingly one comes to this conclusion, for the old Lamarckian school, of which Herbert Spencer is the ardent apostle, held out the promise of great things. What might we not achieve by the continued inheritance of beneficial acquisitions? But this dream, so fondly clung to by our grand old thinker, is apparently but a dream, and it would seem that we must look for progress, not by direct equilibration, as he terms it, but by indirect, in other words by natural selection. Now this selection is of two kinds (*a*) sexual, of which I do not propose to say anything here save that the whole question of marrying is one of proper sexual selection, and (*b*) natural selection commonly so-called, or the survival of the fittest. Since animals tend to increase in geometric ratio, and since there is only room for a certain number, a struggle for existence ensues resulting in the survival of the most fit. Any animal possessing an attribute giving it an advantage over its fellows tends to be selected, and seeing that this beneficial attribute tends to be inherited, it is clear that a species may in this way be considerably modified in the course of several generations, just as happens in the case of the artificial selection practiced by breeders of domestic animals.

The tendency of organisms to increase in geometric ratio is not, however, the only cause of natural selection. In the first place the environment is always changing, and this involves the continued readjustment to it of successive generations, by a survival of those individuals varying in suitable directions. But even were the environment unchanging, and were there no geometric increase, natural selection would still be needful in order to maintain adaptation owing to the law of deterioration. Natural selection, in short, has not done its work when it has achieved adaptation to a certain environment; its continued operation is needful in order to maintain a certain level of excellence, otherwise racial deterioration must ensue. Natural selection thus operating has been termed "panmixia." The offspring of even the most perfectly adapted parents are not all alike, but vary round a certain standard of excellence. Some fall short of it, and these require to be weeded out if the standard is to be maintained. Take as an instance the case of eyesight. In order to maintain this at a high standard there must be a continual elimination of individuals with defective sight. Among savage peoples even moderate degrees of short sight place the individual at a great disadvantage in the hunt and the fight, and tend to his premature death, thus preventing him from leaving progeny with like defect. And what is true of the organs of sight applies to all the organs of the body—they are all liable to fall short of the standard of excellence, and panmixia is needful to the maintenance of that standard.

The tendency of species to increase in geometric ratio, the changeableness of the environment from generation to generation, and the occurrence of variations which fall short of the standard of excellence—these, then, are the factors which start the operation of natural selection. The first of them has little direct effect upon selection among civilized communities, since in their case food is placed within the reach of practically all, and those unable to procure food for themselves do not die as they would under more primitive conditions; but elimination from other causes is still busily at work among us. *Every death not due to accident or old age is really the elimination of an unfit variation*, and occuring before the end of reproductive life falls under the category of natural selection.

A word as to elimination due to changing environment. We are surrounded by vast armies of pathogenic organisms. Did they but remain unchanged we should sooner or later become adapted to them (as we should to any environment not fatal to the entire race) by the continued elimination of those of us least able to resist them; but these organisms being living are ever varying like ourselves and adapting themselves to new conditions, and consequently we never can become adapted to them all. We may possibly succeed in becoming adapted to some species and in starving out others, but depend upon it mankind will always be liable to germ diseases, let nature strive how she may to bring about adaptation to the organisms causing them, and we may be sure that untold millions will be sacrificed in the attempt.

Next as to elimination by panmixia. The wonder is, if we come to think of it, not that it should operate, but that its operation should not be much more stringent than it actually is. Think for a moment of the marvellous complexity of the human organization. Take any limited branch of physiology, make a life-long study of it, and every day will increase your wonder at the intricate adjustment of means to ends. Whether we study the physiology of the heart and vascular system, of phagocytes, or of sugar metabolism, or whether we contemplate the mysterious cerebral processes which underlie the operations of mind, we shall be equally impressed with the fact; and when once imbued with it we can only marvel that our hearts and vaso-motor systems, our phagocytes, sugar-forming organs, and brains serve us so well as they do, and that more of us do not suffer from palpitations, flushings, defective phagocytes, diabetes, and insanity than is actually the case. How is this high level of excellence maintained? The answer is, by panmixia. If panmixia is needed in order to keep the several parts of the eye so nicely adjusted as to render accurate accommodation possible, must it not also be necessary in order to maintain the same level of excellence in respect of the cardio-vascular system, the phagocytes, and the tissues which subserve the glycogenic and kindred functions? As a matter of fact, defects in these systems constantly occur; a certain number of people do get serious heart disturbances, do get diabetes, do suffer from defective phagocytosis, and do go mad, and all such tend to get eliminated by the action of panmixia. The defects may, however, not be sufficiently great to lead to death. Many suffer from wretched health even when placed under the most favorable conditions. I am thinking especially of the large army of neurotics and hypochondriacs who, though life is but a misery to them, yet do not die and who thus escape the operation of panmixia. Nevertheless, it cannot but be supposed that under more primitive conditions it would operate among them.

This leads me to refer more at length to the interference with the operation of natural selection among civilized communities, for though, as I have said, natural selection is actively operating among them, it is of course to a large extent interfered with. Many lives are saved which would otherwise succumb through incapacity to cope with the environment. If every individual had to provide for himself, a large number would succumb who now live by the sufferance of their fellows. Some would succumb through not having succiently keen senses—hearing, eyesight; others through being crippled in their limbs; more from general weakness; others again from strangulated hernias, ovarian cysts, and so forth. These are now in numberless cases saved, and are thus enabled to have children who tend to inherit the like defects. Hence it follows that these defects are continually increasing among the civilized, and that the level of physical perfection among them is lower than among primitive communities, and if the present state of things continues, it must go on getting lower still.

I am tempted here to refer briefly to another interference with the operation of selection among the civilized. Among primitive communities polygamy generally prevails. Those members of a savage tribe who by their mental and physical superiority are able to raise themselves to the rank of chiefs, secure the largest number of wives and leave the largest number of children to inherit the paternal excellences. Now while as a sociologist I am dead against polygamy, as a biologist I confess (at the risk of being accused of regarding humanity from the point of view of a stud-groom, as some one has put it) I cannot but regret its complete abolition. I would like to see our giant intellects and champion athletes leave a numerous progeny.

To turn now to the question of heredity. It is very necessary that we should understand its potency. The structure of an individual depends almost wholly upon it. The power of natural selection to mould a species presupposes its influence, for it assumes that the individual "selected" will transmit his peculiarities to his offspring. Let us ever remember that what we are, whether fair-skinned or dark-skinned, long-nosed or short-nosed, big-brained or little-brained, depends essentially upon our parentage. The influence of heredity in disease is vast, much greater, in my opinion, than most medical men deem it to be. In the text-books we are told that one disease is hereditary 30 per cent, another 10 per cent, while a third disease is not hereditary at all. Not hereditary? I maintain that all diseases are hereditary; *you cannot eliminate the influence of heredity* from any disease, and I will prove it. What is disease? It is abnormal life. And what is life? It consists of an interaction between the individual and his environment; therefore disease is an abnormal interaction between the two. It follows that you cannot possibly eliminate the part played by the individual from the causation of any disease, since it is *his structure which determines whether he shall or shall not react abnormally to a given environment.* Now his structure is essentially determined by heredity. I have therefore proved my point. This is no mere logical quibbling, but a practical fact. I will test my position by taking a disease with which most people will probably say heredity has nothing at all to do—the wheal caused by flea bite. This affection depends upon the morbid interaction of the host and the specific environment constituted by the poison with which the parasite inoculates him. As everyone knows, the liability to be flea bitten and the tendency to suffer from wheals when thus bitten, differ greatly in different individuals. Some suffer little or not at all in this way; others grievously. Upon what does this difference depend? It depends upon differences in the organization or structure of the host, and these differences are essentially determined by heredity. Hence all parasitic diseases, from a flea bite to pulmonary consumption, from typhoid to malignant tumor, are hereditary. Such diseases depend upon defect in the organism as regard some specific environment, but others, such as chronic Bright's disease, diabetes, ovarian cyst, strangulated hernia, may occur independently of any specific environment, and it is obvious that these are *a fortiori* hereditary.*

To reiterate briefly the chief conclusions we have arrived at. There seems good reason to believe that organic evolution proceeds essentially by a survival of the fittest, and that all further evolution of man must take place essentially by the operation of this process. Natural selection is needful not only to *attain* adaptation of the individual to his environment, but also to *maintain* it. Structure is chiefly determined by heredity, and seeing that disease is a morbid interaction between the individual and his environment, and that his structure determines whether he shall or shall not react abnormally to a given environment, it follows that the factor of heredity cannot be eliminated from the causation of any disease.

We are now in some sort of a position to answer the question: Who are unfit to marry and have children? A question I can only very briefly answer in a paper like the present. We have seen that panmixia is largely interfered with in civilized communities, with the result that racial deterioration must inevitably be in progress, and will disastrously continue unless some new means is discovered by which man's present level of adaptation to his environment can be maintained. Happily, we have such a means to hand, and it is from among those very forces of progress wherein, as we have seen, arises his danger, that his safety also is to be found. For the leavening influence of high ideals for nearly 2000 years has had its effect, and modern progress, modern civilization, tend more and more consciously or unconsciously, and whether we recognize it or not, towards altruism; it has already widened out man's conception of the duty he owes his neighbor, until that duty has come to mean, to those at least who "feed the high tradition of the world," the service of humanity at large, and more enlightenment as to his undoubted responsibility to posterity will show him that that service does not end with the present generation, but reaches forward to a future to which we can set no limits. Fortified with the conviction that generations yet unborn have also a claim upon him, a man could of his own free choice do far more than the blind working out of natural laws has ever done, or ever can do, to stamp out the tendency to disease and to perpetuate a

---

*I have discussed the influence of heredity in disease in my work, "The Causation of Disease," p. 318, et seq.

healthy race. For all that is needed to enable panmixia to proceed as beneficially as ever, and that without the ruthless destruction of the unfit which obtains when natural selection works unchecked, is for those which have defects *which under natural conditions would lead to annihilation* to forbear to procreate. As beneficially, did I say? Nay, very much more beneficially, for not only could those who are already marked by disease refrain from marrying, but those also who, as judged by their family history, are likely to become ultimately the victims of serious disease. And if it be urged that such a self-denying ordinance presupposes a higher ethical development than the generality of men have at present attained, that by no means disproves my contention; it only proves that much has yet to be done to get a sound public opinion, a sensitive public conscience on these matters, such an opinion and such a conscience as I hold it to be one of our first duties to strive to create. Meantime, I doubt very much whether the renunciation would be felt to be so very great if men's minds were trained to realize all the issues that are involved, and if it were plainly shown, as it might be, that the sum total of human happiness would be increased by it. What I do recognize is that it must take time for society to get out of the old grooves of thought, sanctioned by almost the whole of our current fiction, as to marrying and giving in marriage, and that while it is getting out of them there must be at times personal suffering, stress and conflict where strong feeling is involved. But all progress is obtained at some sacrifice, the first steps in a reforming movement nearly always cost someone dear, and I can imagine a state of society gradually evolving out of this transition period in which there shall be such a sound healthy view of the responsibilities of marriage that the idea of any union that would be at all likely to involve the survival of the unfit would not be entertained for one moment, and where consequently, there would not be felt to be any renunciation to make, any more than there is now in keeping within the prohibited degrees of the prayer book and not marrying one's grandfather or grandmother. It is a notable and I think hopeful sign of the times that in a recent novel by Robert Buchanan, in which the reader is introduced to the twenty-first century, he assumes that by a careful elimination of the unfit a state of physical perfection has been achieved. The markedly unfit are disposed of in the chamber of Euthanasia, and man and woman may not "under any circumstances come together in lawful wedlock without a certificate of physical perfection from the Holy Office of Health." Now though the time is far yet when men will submit to State coercion in such matters, it is not therefore, too soon to urge their responsibility upon them, and everything that will awaken them to it, whether from the press or the pulpit, is to be welcomed.

Let us now consider under what circumstances the physician ought to use his influence to prevent marriage. In certain cases there can be no doubt as to the course he should take. Such serious diseases as pulmonary consumption, organic heart disease, epilepsy, insanity, diabetes, and chronic Bright's disease, are obvious bars to marriage. Not only the present existence of any one these of diseases, but the having had it and recovered from it would, in my opinion, constitute unfitness. The same restriction applies, I think, to rheumatic fever (certainly if there has been more than one attack) and to most cases of acute Bright's disease not due to scarlet fever, especially when the inflammation has been of more than two months duration.

There are other disorders equally serious in themselves, but the having suffered from which is not usually regarded as a bar to marriage. I allude to all those cases of non-accidental disease in which life is saved by the surgeon's skill. Most, if not all, individuals of this class should be regarded as unfit for procreation. Take the case of a person with strangulated hernia or ovarian cyst. In a less artificial condition of existence such a one would be weeded out as unfit and thus prevented from transmitting to others the like defect. Let us use all the means at our command to rescue such from death, but it must be upon the clear understanding that no children shall be born to them afterwards. Think of the millions and millions of women suffering from ovarian cyst, whom nature has in the past sacrificed with a cruel kindness, in order to keep up a certain level of ovarian fitness in the race. These are practically all saved now in civilized communities, with the result that the percentage of ovarian cysts is necessarily increasing. In can only be because medical men do not realize the potency of heredity that they sanction the propagation of children by women whose lives have been saved in this way.

In cases of the class just referred to there can be no doubt about our course, but in other and kindred cases we may find it more difficult to decide what to do. We cannot rely on the rule—in the main a safe one and which I believe should always form the basis on which our own judgment rests—that those with some defect which under primitive conditions would lead to destruction should refrain from marriage. To take an example, a person with a bad error of refraction can be made to see well with glasses and may become a very useful citizen, and although we cannot but regard with regret and misgiving the increasing defect of vision taking place in consequence of the interference with panmixia in this one single class of cases only, it would need more boldness than most of us possess to advise a person to refrain from marrying on account of such defect alone. We should be merely laughed at. With congenital defect of hearing the case is different. There we can speak with authority: none such should procreate.

I now come to a particular class of disorders which I think should beyond almost all others be regarded as a bar to marriage. I mean functional disorders of the nervous system. The highly sensitive are not suited for this hard word. Its strenuous condition calls for men with iron nerve and stout hearts. I fear it must be acknowledged that as regards happiness—which at any rate from the purely physical point of view may perhaps be fairly looked upon as our being's end and aim—the fine animal with little imagination and a good thick skin has the best time of it here—is the most truly "fit." It may be that such a one cannot rise to the same heights of happiness as his more nervous and imaginative brother; but, on the other hand, he is incapable of sinking into the same depths of wretchedness. The future has no terrors for him, for he has no imagination to depict it; he lives in the enjoyment of the present, and even when afflicted with the gravest organic disease he suffers very little as compared with people of weak nerves, whose lives are often one long-drawn-out misery. In this latter class I include not only those who suffer from continual headache, noise in the ears, depression of spirits, and so forth, but the hysteric and hypochondriac class also. To the neurotic man as well as to all of these I should say, "remain single." He is often advised to marry on the ground that the wife and children will draw him out of himself, and so far the advice is doubtless good, but I fail to see what right we have to sacrifice the prospective wife and children. I have seen too much home misery caused by neurotic parents to connive at such an arrangement.

In testing an individual's fitness to marry we have to consider not only his past history and his present state, but his probable future as judged by his family history, and this enables us, as already observed, to be more searching than even natural selection. Among the many evils which it would be possible greatly to diminish, if not eradicate, by obeying the warnings furnished by the family history are apoplexy and premature senilty. Seeing that deaths from these causes do not as a rule occur until after the procreatic age—i. e., until after the individual has had the oportunity of leaving a full complement of children—such deaths leave little direct impress upon the race. In regard to the staving off of senility I hold it to be well within our province to attempt to do so. The fate of the man in his sixties who, in the zenith of a sccessful career, or having just settled in life, is struck down by apoplexy, or who suffers from a general senile breakdown, is surely a tragic one, and I contend that we should wage hot warfare against such occurrences. Some there are whose blood-vessels degenerate, whose hair turns gray, and whose tissues generally show a tendency to senility while yet in their thirties; and on the children of such, especially those who "take after" the prematurely senile parent, we should for the most part urge abstention from marriage. For it is quite remarkable how individuals as they grow old imitate the old age of their parents, sometimes of the one, sometimes of the other. How often does it happen that the young have occasion to criticise the testiness, the ill-nature, or the selfishness of the old. It seems to them quite impossible that they can even be the same themselves, and yet as the years roll on it too often happens that all the weakness of the parent recurs with painful exactitude in the child. Indeed, the exactness with which the individual repeats the life history of his progenitors is freshly impressed upon me every day of my life and I often find myself involuntarily forecasting the future of the child by reference to the condition, mental and physical, of the parent. I have in my mind a case in point. Mr. and Mrs. A. are between forty and fifty years of age. Each became decidedly gray before thirty, and as in all such cases each is highly neurotic and has within recent years developed several nervous symptoms. Mr. A., indeed, shows signs of a complete nervous breakdown. Now there are seven children of this marriage from eighteen years of age downwards and they are what would be called remarkably

"fine" children; yet it is as certain as anything can well be that the majority of them, if not all, will, like their parents, become prematurely gray and neurotic. To look at them now without any reference to their family history, no one would anticipate this for a moment, but judged in that light it is no mere idle fancy, but almost a certainty; and if we were able to prevent marriage in their cases we should be helping towards the eliminating of a prematurely senile and neurotic type.

To return, in conclusion, to what I have already touched upon—the difficulty of a practical application of these views. As a general reply to the objections that few are capable of the sacrifice of enforced celibacy where the affections are engaged, and that any interference with marriage on the grounds above given is an undue infringement of the liberty of the subject, I would observe that the life of the individual living in a civilized community is one long continued self-restraint, and that each such individual has very much less freedom of action than is generally supposed. Turn where he may he will find his action restricted, for, being part of a highly complex social machinery, elaborate rules of conduct are necessarily imposed upon him; mere prudence indeed, compels a man to constantly restrain his natural impulses. And surely when it is realized what are the tremendous issues at stake we may take a higher ground than the ground of mere expediency and appeal to the sense of duty; but it was a very poor and inadequte conception of duty which could content itself with the fulfillment of obligations to the living, heedless of those who shall come after. Theft and murder are considered the blackest of crimes, but neither the law nor the church has raised its voice against the marriage of the unfit, for neither has realized that worse than theft and well-nigh as bad as murder is the bringing into the world, through disregard of parental fitness, of individuals full of disease-tendencies. This has been left to our own profession, and we shall be unworthy of its traditions if we do not, each of us in his own particular sphere, strive to bring nearer the day when, not in a heritage of woe, but of blessing, the deeds of the fathers shall be visited upon the children unto the third and fourth generation.—*London Lancet.*

Wimpole Street, W.

